# Need for mechanically and ergonomically enhanced tremor-suppression orthoses for the upper limb: a systematic review

**DOI:** 10.1186/s12984-019-0543-7

**Published:** 2019-07-18

**Authors:** Nicolas Philip Fromme, Martin Camenzind, Robert Riener, René Michel Rossi

**Affiliations:** 1 Empa, Swiss Federal Laboratories for Materials Science and Technology, Laboratory for Biomimetic Membranes and Textiles, Lerchenfeldstrasse 5, 9014 St. Gallen, Switzerland; 2 Sensory-Motor Systems Lab, Department of Health Sciences and Technology, Institute of Robotics and Intelligent Systems, ETH Zurich, Tannenstrasse 1, TAN E 5, 8092 Zurich, Switzerland

**Keywords:** Tremor, Suppression, Wearable, Upper limb, Orthosis, Device

## Abstract

**Introduction:**

Tremor is the most common movement disorder, affecting 5.6% of the population with Parkinson’s disease or essential tremor over the age of 65. Conventionally, tremor diseases like Parkinson’s are treated with medication. An alternative non-invasive symptom treatment is the mechanical suppression of the oscillation movement. The purpose of this review is to identify the weaknesses of past wearable tremor-suppression orthoses for the upper limb and identify the need for further research and developments.

**Method:**

A systematic literature search was conducted by performing a keyword combination search of the title, abstract and keyword sections in the four databases Web of Science, MedLine, Scopus, and ProQuest. Initially, the retrieved articles were selected by title and abstract using selection criteria. The same criteria were then applied to the full publication text. After the selection process, relevant information on the retrieved orthoses was isolated, sorted and analysed systematically.

**Results:**

Forty-six papers, representing 21 orthoses, were identified and analysed according to the mechanical and ergonomic properties. The identified orthoses can be divided into 5 concepts and 16 functional prototypes, then subdivided further based upon their use of passive, semi-active, or active suppression mechanisms. Most of the orthoses concentrate on the wrist and elbow flexion and extension. They mainly rely on rigid structures and actuators while having tremor-suppression efficacies for tremorous subjects from 30 to 98% using power spectral density or other methods.

**Conclusion:**

The comparison of tremor-suppression orthoses considered and mapped their various mechanical and ergonomic properties, including the degrees of freedom, weight, suppression characteristics, and efficacies. This review shows that most of the orthoses are bulky and heavy, with a non-adapted human-machine interface which can cause rejection by the user. The main challenge of the design of an effective, minimally intrusive and portable tremor-suppressing orthosis is the integration of compact, powerful, lightweight, and non-cumbersome suppression mechanisms. None of the existing prototypes combine all the desired characteristics. Future research should focus on novel suppression orthoses and mechanisms with compact dimensions and light weight in order to be less cumbersome while giving a good tremor-suppression performance.

## Introduction

### Definition and prevalence of tremor

Tremor is defined as a rhythmic and involuntary oscillating movement of a body part [1]. It is the most common movement disorder in adults, and may develop as a consequence of disease or drug use [1, 2]. Of the two most prevalent conditions causing tremor in the upper limb, the disease essential tremor (ET) is prevalent with 4.6% of the population aged 65 or over and 21% older than 95, whilst Parkinson’s disease (PD) is known to develop in 2% of all people older than 65 [3–7]. For those who develop PD, tremor manifests in 50% of those afflicted with the condition, whereas symptomatic tremor is ubiquitous in ET [7]. In both ET and PD, the hands are the most affected site of the body regarding tremor [5, 8]. With the total prevalence of ET and PD in the population over 65 years, 5.49 million people in the European Union are diagnosed, accounting for more than 1.1% of the population of the European Union [9]. In addition, it is forecast that the demographic change will lead to an increasing number of tremor patients [9]. More than 65% of the population with upper limb tremor show serious problems in performing their activities of daily living [8, 10]. This can additionally lead to social exclusion. Furthermore, 48% of the PD patients and 34% of the ET patients are at least mildly depressed due to their lack of performance in activities of daily living [11].

### Different tremor types

In the upper limb, different tremor types can occur. Rest tremor appears in the affected body part when it is not voluntarily activated, whereas action tremor occurs with voluntary activity of the limb. With action tremor, the two main subgroups are postural tremor and kinetic tremor. Postural tremor appears while maintaining voluntarily a position against gravity, whereas kinetic tremor occurs during voluntary movements. [1] The main symptom in ET is action tremor in the upper limb, especially kinetic tremor, whereas rest tremor is typical for PD [12, 13].

### Treatment

The exact cause of ET and PD is unknown [14, 15]. Neither of these diseases is curable and the treatment is focused on relieving the symptoms [16]. The most commonly used treatment for tremor is medication, although up to 53% of people discontinue medical treatment due to side-effects or lack of efficacy [17, 18]. Several surgical options for tremor treatment exist, including radiofrequency lesioning and gamma knife radiosurgery. Deep brain stimulation (DBS) as a surgical procedure is the most effective treatment for most tremors and is applied for advanced and selected cases [3]. Deep brain stimulation is, however, an invasive treatment with the potential for adverse events, such as cognitive, psychiatric and behavioural status change, that affect up to 48% of the people undergoing the surgery [19]. The tremor reduction efficacy of medication ranges from 23 to 59% for PD [20] and 39 to 68% for ET patients, whereas DBS has a tremor reduction of 90% [3]. In the study by Koller et al., 16% of the patients had a loss of efficacy of the DBS within 40 months [21]. Although there is a lack of long-term pharmacological studies [21], Sasso et al. showed that the Primidone tremorolytic effect only lasts for up to 1 year [22]. A new emerging surgical treatment of tremor is high intensity focused ultrasound. A recent study showed that this less-invasive method reduced the tremor score by 55% after 6 months, which is also related with mild to moderate adverse events [23]. More precisely, 74 neurological adverse events in 56 patients were observed, while an alteration in sensation was the most common one in 38% and persisted at 12 months in 14% [24]. Alternative treatments are required for patients not responding to medication (50% of ET), who are drug intolerant or are not suitable for deep brain stimulation [25]. Due to these side-effects and the lack of efficacy, there remains a need for non-invasive treatments. Even with optimal medical or surgical intervention in tremor, patients will still require physical interventions and occupational therapy to promote social participation [26]. Adding weight to the limb, limb cooling, vibration therapy, transcranial magnetic stimulation, sensory electrical stimulation, and functional electrical stimulation are current alternative and supplementary treatments of tremor [17]. An emerging alternative and supplementary treatment is the physical intervention and suppression of the occurring oscillating rhythmic movement with a wearable external orthosis.

### Purpose of review paper

The purpose of this review is to identify the scope for improvement of past wearable tremor-suppression orthoses for the upper limb in order to improve future orthoses. In particular, we aim to investigate relevant mechanical properties, including the impact on ergonomics, analysing the advantages and disadvantages of current orthoses. Apart from general papers reviewing wearable and robotic rehabilitation devices, to the best of our knowledge, no such review has been performed concentrating on the mechanical and ergonomic properties of tremor-suppression orthoses.

## Biomechanical background

### Anatomy of the upper limb

An understanding of the biomechanics is necessary to define the necessary specification of a wearable tremor-suppression device. It is essential for the degrees of freedom (DOF) in the anatomy of the upper limb for wearable devices to match the inherent mobility of the limb and avoid inhibiting the movement of the user. Not considering the DOF in the hand, the arm has 7 DOF from wrist to shoulder: flexion and extension (WFE), and radial and ulnar deviation (WD) in the wrist, pronation and supination (FPS) in the forearm, flexion and extension (EFE) in the elbow, and flexion and extension, abduction and adduction as well as internal and external rotation in the shoulder. [27] Besides the biomechanics of the upper limb, it remains essential that the location of ligaments, blood vessels, nerves, and tendons is considered in the design of a wearable device to prevent unintentional harm.

The kinematics of the arm and thereby those of tremor, like joint inertia and joint stiffness, are changed by a wearable assistive device, for example by its weight. Furthermore, the physical suppression of tremor can cause a shift of tremor from the suppressed distal joint to a proximal joint (defined as the Distal to Proximal Tremor Shift phenomenon), observed in one out of six patients in the study by Manto et al. in 2007 [28].

### Characteristics of tremor

Tremorous movement types are usually characterised by their frequency. Several frequency definitions have been published for the two most common tremor diseases ET and PD, ranging from 4 to 12 Hz for ET and 3 to 10 Hz for PD [4]. For both ET and PD tremor, the tremor frequency decreases with time over the course of the disease [29]. The intensity of the tremor is also dependent on the progress of the disease. Only a few investigations on the biomechanical processes of tremor in the upper limb and the generated forces, especially during activities of daily living, have been accomplished [30, 31]. These forces are important parameters to design a suppression system. Such an orthosis needs to apply an adequate counter-force in order to suppress the involuntary movement.

The visual feedback of the limb with a reduced tremor amplitude when using a wearable tremor suppression device leads to a positive impact on the human control system, reinforcing the human motor control and further reducing tremor [32]. The mind-set of a tremor-affected person is of importance, as anxiety or emotional stress, for instance, can intensify tremor [15]. In this context, it can be assumed that a good acceptance of the device by the user leads to a better mind-set and better tremor suppression respectively.

### Tremor orthoses in activities of daily living

Wearable assistive devices for activities of daily living should be worn and used through the entire day. Therefore, an ergonomic design is one of the biggest challenges in such devices to ensure an adequate performance [33]. The ergonomic design is influenced by weight, thermal and skin sensory comfort (human-machine interface), restrictions in DOF, and resistance forces for voluntary movements. Comfort includes sufficient heat and moisture (sweat) transport as well as the avoidance of excessive friction, shear loads and normal loads. Tremor results from interaction between pathological neural control and the frequency response of the limb, which means that the change of joint stiffness by adding weight, for instance, can change the frequency response and therefore result in milder tremor [34, 35]. However, the weight and size of the orthosis influence the ergonomic design, such that it is preferable that the mass should be as lightweight and the size as small as possible. In a study with 242 users of upper limb prostheses, the weight was revealed to be one of the most important design factors, as for neurological patients the additional weight led to fast fatigue [36].

## Methods

### Search strategy

**Fig. 1:**
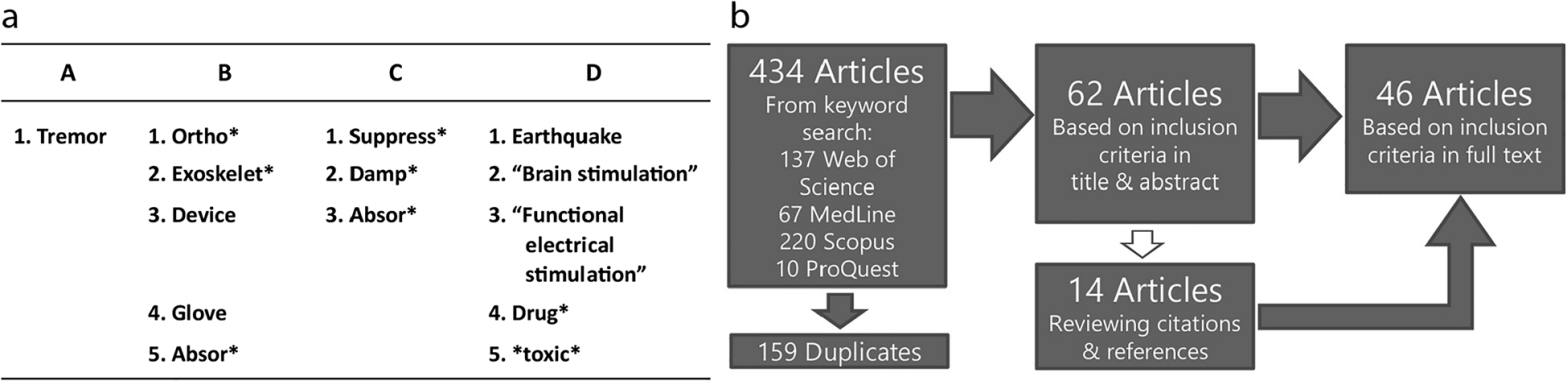
Database search and paper selection. **a** Keywords for database search are grouped in sets. Within a set the “OR” function is applied, whereas the sets A, B, C are connected by “AND” and set D by “AND NOT” function. The symbol “*” refers to a truncation symbols in the database. **b** Literature selection process and results from retrieving articles from the databases to the selected papers, based on the criteria

A literature search was performed by a combination of specific keywords found in the title, abstract and keyword section of the papers. These keywords were grouped by their synonyms and combined with Boolean operators (see Fig. 1). Furthermore, truncation symbols to create searches with various endings were used to ensure that all relevant articles were included. For the intra-set function the “OR” operator and for the inter-set function the “AND” operator were used. Exclusion criteria were collected in set D (Fig. 1) and used with the “AND NOT” function. Further criteria in the database search were “English” as article language and no limitation of the publication date. These search parameters were used in the four databases Web of Science, MedLine, Scopus, and ProQuest. 434 articles were retrieved on August 13th 2018.

### Paper selection

After retrieving the primary search results from each database, the following inclusion and exclusion criteria were applied to the selection and rejection of the papers to narrow down and gain the desired literature:

- Inclusion
  - An orthosis applies an active or reactive force to the human skeletal system
  - The orthosis suppresses physiological tremor
  - The orthosis treats upper limb tremor
  - Wearable orthosis/exoskeleton
  - Prototypes and concepts
- Exclusion
  - Functional electrical stimulation is used for milder tremor
  - Drugs are used to decrease tremor
  - Surgery is used to decrease tremor
  - The paper is focused on the control algorithm
    (except if it contains the control of an included device)
  - Hand-held devices
  - Table-mounted devices
  - Suppression of finger tremor

All search results retrieved from the databases were saved into EndNote X8 reference manager software (Clarivate Analytics, Philadelphia, United States) for the selection process, removing the duplicates (see Fig. 1). After retrieving the 434 articles, the selection process was conducted regarding the following steps: 1) Computerized removal of duplicates; 2) Evaluation and selection of the articles by title and abstract considering the inclusion/exclusion criteria; 3) Retrieving new articles from cited article list and reference list while excluding duplicates; 4) Evaluation and selection of articles by full text considering the inclusion/exclusion criteria.

Following the selection process, the devices described were sorted by their development status, whereupon they were classified into concept and prototype. A concept is defined as a theoretical design not exceeding the model validation in the development process, whereas a prototype is defined as a functional device with the possibility to validate and evaluate its properties.

The orthoses were classified by the type of the vibration suppression: passive, semi-active and active. Semi-active and passive technologies suppress involuntary movements, whereas active technologies suppress involuntary while supporting voluntary motions of the wearer’s limb. More specifically, passive suppression comprises robust methods regarding the design which are dependent on constants like a spring and damper coefficient. A passive spring-damper system has a low resistance force at low and a high resistance at high velocities. Slow and deliberate movements can be accomplished, whereas fast movements create a higher reaction force. Semi-active orthoses work like passive orthoses but use tuneable mechanism characteristics, regulated by a controller. An active orthosis reacts to sensory information with an actuator. An equally strong, oppositely directed force is used to counteract the involuntary movement.

### Framework of comparison

In this review different orthoses were compared with the help of a framework of the key design elements of the orthoses. For the investigation of the mechanical and ergonomic properties of the tremor-suppression orthoses, relevant information was collected, stored and analysed systematically. For the quantification of the ergonomic performance, both objective and subjective factors – including performance and comfort – play a role in determining whether a person will rate the orthosis as sufficiently comfortable, convenient and unobtrusive to wear on a regular basis for activities of daily living. These investigations have been focused on quantifiable aspects like the DOF, as an indicator of restrictions in the natural workspace. For this purpose, we defined a DOF coefficient which represents the DOF of suppression per locked and suppressed DOF. DOF of suppression are the number of human DOF of which an orthosis suppresses the involuntary movement, whereas the locked and suppressed DOF include additionally the number of DOF where the orthosis locks movements. The coefficient illustrates how many DOF are suppressed while being connected over a certain amount of DOF. Further, we defined a specific weight as weight per DOF (g/DOF). Since the retrieved orthoses work with varying DOF, the specific weight is used to compare the weight of the orthosis per DOF suppressing involuntary movements. This method compares the weight while considering the additional weight for extra suppression mechanisms and connection structures per DOF.

The comparison of the mechanical properties for this review primarily focuses on the suppression mechanism. One aspect is the prevalence of the different suppression mechanisms. A typical characteristic of active orthoses is the specific power, since in rehabilitation robotics, a high strength-to-weight ratio for actuators is required [37]. The weight of the supporting structures and attachments, as well as the energy supply like batteries is not included, since these are usually considered independently from the actuation system. The mechanical characteristics of the human skeletal system are used as a benchmark. For the semi-active and passive orthoses, the suppression characteristic is given by the damping coefficient and for active orthoses the force and torque, respectively. The efficacy of the orthosis to suppress the involuntary movement is the determining factor of its performance. Here, the efficacy is the capacity of a beneficial change of tremor by the orthosis. More precisely, the reduction of the tremor power (power spectral density - PSD), root mean square of the tremor acceleration amplitude (RMS), average tremor acceleration (AA) or deflection amplitude (AD) are here the beneficial change. Regardless of the different methods being employed, it can be assumed that the resultant effect on efficacy is similar or of an equal range, as power, acceleration, and deflection depend on each other. An average of the different efficacy methods (cross-method efficacy) will therefore be analysed. The efficacy in this review is given as a percentage and has been converted for the orthoses using a decibel ratio. Only the first, dominating harmonic frequency was considered in this review. For papers reporting a range of efficacy for the orthoses, the average value was calculated. All in all, the following elements of the orthoses are reviewed:

- Suppression efficacy
- Degrees of freedom
- Specific weight
- Specific power and force/torque (active)
- Damping coefficient (semi-active and passive)

## Results

Based on the inclusion criteria, the results of this literature review paper contain 46 of 434 publications which were identified on August 13th 2018. The literature search resulted in a total of 21 suppression orthoses (16 prototypes and 5 concepts), numbered from 1 to 21 in Tables 1 and 2. Some exemplary orthoses are shown in Fig. 2. The main difference between the devices is the suppression type (active #1-#8 and #17-#19; semi-active #9-#12, #20 and #21; and passive #14-#16).

**Table 1:** Orthoses prototypes for tremor suppression. Information not provided is marked with “?”

#	Orthosis name/Group	Supp. type	Suppression mechanism	Suppression characteristic	Efficacy [%] (method)	Voluntary movement disturbance	Evaluation method(tremor disease or simulation)	DOF coefficient	Total weight /one attenuator [kg]
1	Voluntary Driven Orthosis Hernstadt [38–40]	active	Direct drive motor [38–40]	3 Nm [38–40]	99.8 (PSD) [39, 40]	0.15% (magnitude change) [39]	test bench (1 ET/PD dataset) [38–40]	1/1 (EFE) [38–40]	0.875 / 0.334 [39]
2	EMG Exoskeleton Fujie [41–44]	active	Direct drive motor [41–44]	?	?	10.5% (not recognised movement) [42]	?	1/1 (EFE) [41–44]	0.330 /? [42–44]
3	EMG Exoskeleton v2Fujie [45, 46]	active	Direct drive motor [45, 46]	1.3 Nm [45]	50–80 (AA) [45, 46]	no voluntary movement detection yet [45, 46]	1 tremor subject (ET) [45, 46]	1/4 (EFE) [45, 46]	0.410 /? [45]
4	WOTAS Pons/Rocon [28, 47–55]	active	Direct drive motor [28, 47–55]	8 Nm [53, 54]	40 (PSD) [28, 47, 49, 52, 54, 56]	80 mNm (resistance torque) [53, 54]	10 tremor subjects (ET, PD, MS, post-traumatic, and mixed tremor) [47, 51, 53, 54]	3/4 (No WD) [28, 47–56]	0.850 / 0.165 [51, 55, 54, 56]
5	ADL Exoskeleton Imperial College [57]	active	Direct drive motor [57]	2.6 Nm [57]	77 (AA) [57]	?	6 healthy subjects (simulating tremor) [57]	2/3 (WFE, FPS, no WD) [57]	0.350 / 0.072 [57]
6	MMS Tendon Glove Western University [58]	active	Tendon [58]	16.2 N [58]	?	12.4% (not recognised movement) [58]	test bench (7 PD datasets) [58]	1/1 (WFE) [58]	? / 0.229 [58]
7	PMLM Zamanian/Richer [59]	active	Linear motor [59]	400 N/(m/s) & 67 N [59]	97.6 (PSD) [59]	0.36 N (resistance force) [59]	test bench (PD datasets) [59]	4/4 (all) [59]	? / 0.315 [59]
8	Pneumatic Actuator Taheri/Richer [60–64]	active	Pneumatic piston-coil [60–64]	15 N [64]	98.8 (PSD) [60, 62, 64]	4.8% (magnitude change) [64]	test bench (10 PD&ET datasets) [62, 64]	4/4 (all) [60–64]	? / 0.378 [60–64]
9	MR Damper Case/Richer [65–70]	semi-active	Magnetorheological piston-coil [65–70]	8.9–187 N/(m/s) [65–70]	96.33 (PSD) [67]	4.47 N (resistance force) [67]	test bench (10 PD&ET datasets) [67]	4/4 (all) [66–70]	? / 0.204 [65–70]
10	EB Orthosis Hernstadt [71]	semi-active	Electromagnetic friction brake [71]	2.2 Nm [71]	88 (PSD) [70]	? (*probably none)*	3 healthy subjects (simulating tremor) [71]	1/1 (EFE) [71]	0.942 / 0.150 [71]
11	Pneumatic Hand Cuff PSG College of Tech. [72]	semi-active	Pneumatic cuff [72]	?	30 (AA) [72]	Yes [72]	1 tremor subject (ET) [72]	2/2 (WFE, WD) [72]	? /?
12	DVB Orthosis Loureiro/Pons [73]	semi-active	Viscous shear resistance [73]	6.5 -25,000 N/(m/s) [73]	98 (PSD) [72]	14.13% (magnitude change) [73]	1 tremor subject (ET) [73]	2/2 (WFE, WD) [73]	? / 0.200 [73]
13	The Viscous Beam Kotovsky/Rosen [74]	passive	Viscous shear resistance [74]	0.002 Nm/(deg/s) [74]	?	Yes [74]	5 tremor subjects [74]	1/2 (WFE, no WD) [74]	0.265 /? [74]
14	Air Dashpot Orthosis Takanokura et al. [74]	passive	Pneumatic piston-coil [74]	1800 N/(m/s) [74]	80 (RMS.) [74]	Yes [74]	1 healthy subject (electrical stimulation) [74]	3/4 (no FPS) [74]	? / 0.037 [74]
15	Vib-Bracelet Israel Institute of Tech. [75]	passive	Tuned mass (spring + mass) [75]	?	86 (AD) [75]	?	test bench [75]	1/1 (FPS) [75]	0.280 /? [75]
16	Damping Orthosis Israel Institute of Tech. [76]	passive	Rotary damper [76]	0.002 Nm/(deg/s) [76]	?	Yes [76]	1 tremor subject (PD) [76]	1/2 (WFE, no WD) [76]	0.200 /? [76]

**Table 2:** Orthoses concepts for tremor suppression

#	Orthosis name	Supp. type	Suppression mechanism	DOF coefficient
17	Piezoelectric Fibre Glove [77, 78]	active	Piezoelectric fibre composites [77, 78]	2/2 (WFE, WD) [77, 78]
18	Dielectric Elastomers Actuator [79]	active	Dielectric elastomers [79]	1/2 (WFE, no WD) [79]
19	Anti-shaker [80]	active	Actuator-moved mass [80]	1/1 (EFE) [80]
20	Magnetorheological Damper [81]	semi-active	Viscous shear resistance [81]	1/1 (EFE) [81]
21	Soft Band Orthosis [82]	semi-active	Tendon [82]	1/1 (FPS) [82]

**Fig. 2:**
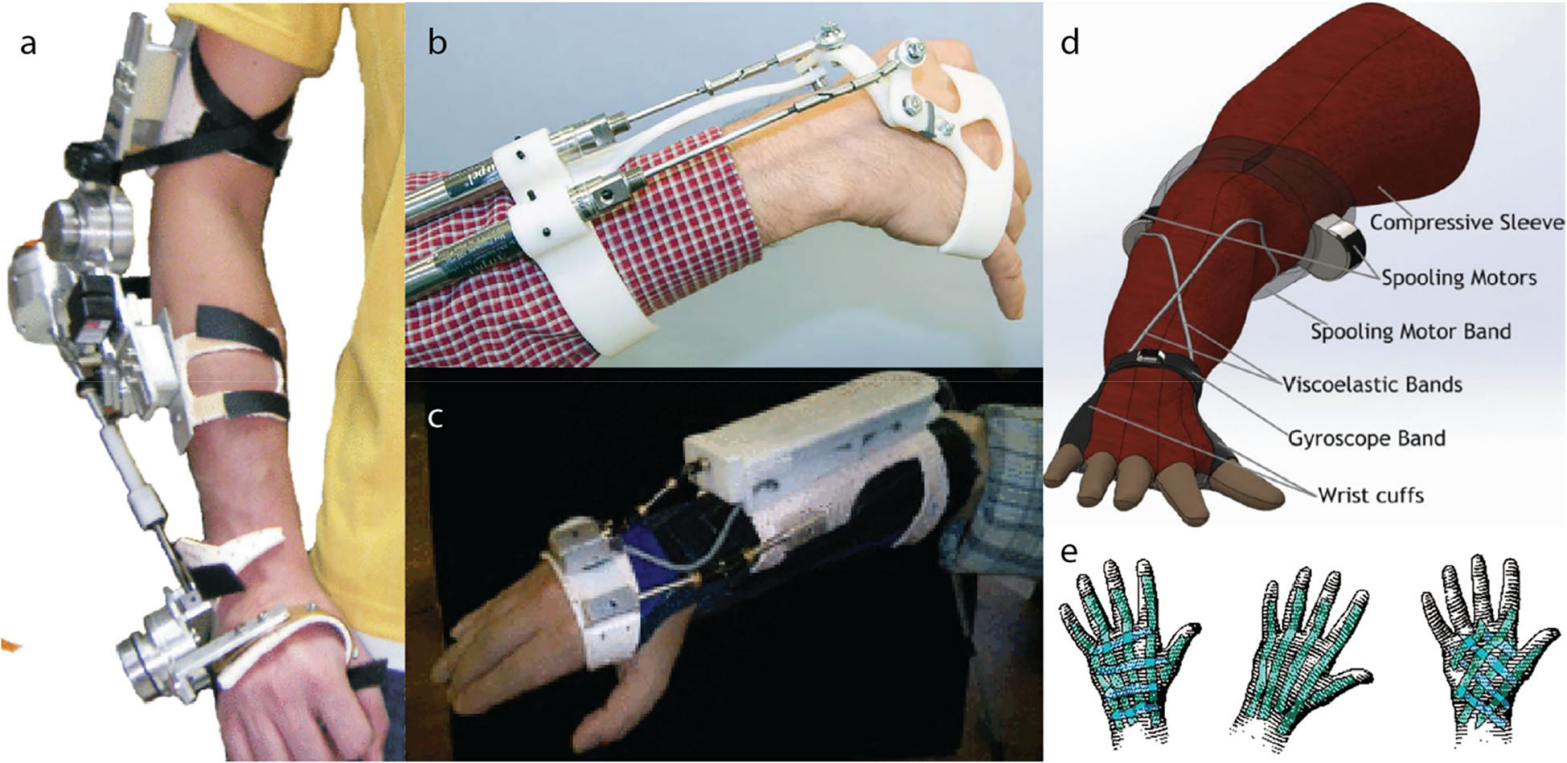
Prototype (**a-c**) and concept (**d**, **e**) orthoses. **a** #4 WOTAS –active attenuator with electrical motor [54]; **b** #8 Pneumatic Actuator –active attenuator with pneumatic piston-coil system [64]; **c** #12 DVB Orthosis –semi-active attenuator using tuneable viscous shear resistance [73]; **d** #21 Soft band Orthosis –semi-active attenuator using viscoelastic tendons [82]; **e** #17 Piezoelectric Fibre Glove – active attenuator with piezoelectric fibre composites [78]. Reproduced with permission from the right holders a, c, d: IEEE; b: Dr. Behzad Taheri, e: Journal of Fiber Bioengineering and Informatics

### Suppression orthoses efficacy

**Fig. 3:**
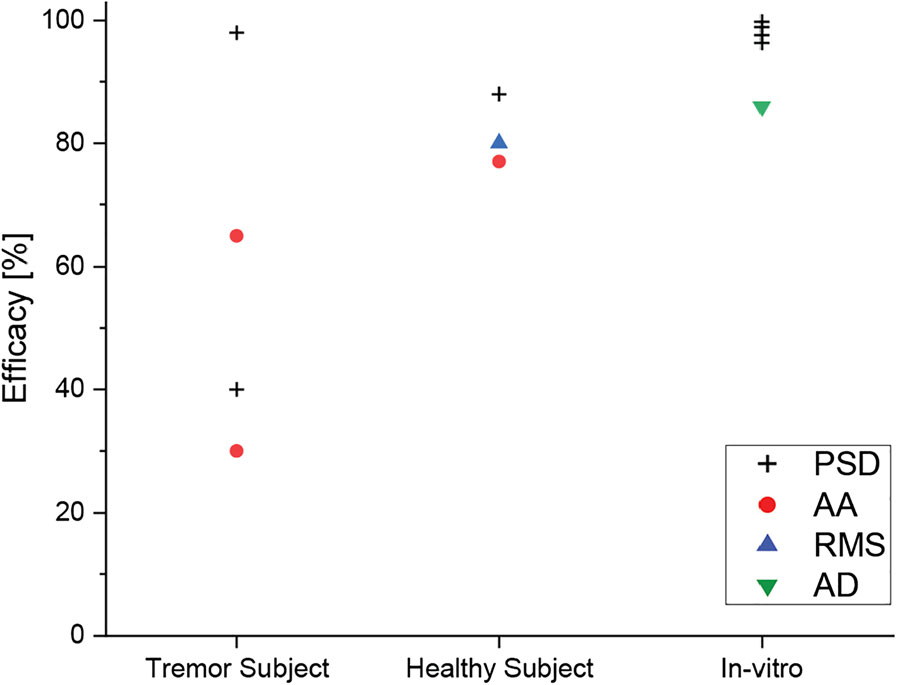
Overview of evaluated performances of the retrieved orthoses. Grouped by evaluation with tremor subjects, healthy subjects or a test bench setup. Analysis methods power spectral density (PSD) labelled with a grey square, average tremor acceleration amplitude (AA) with red circle, root mean square of the tremor acceleration amplitude (RMS) with blue triangle and average tremor deflection amplitude (AD) with an inverted green triangle

The efficacy of the suppression orthosis indicates how well the orthosis counteracts tremor. The performance of the prototypes was evaluated either through test bench experiments, on tremor-affected patients or on healthy subjects, the latter of whom were treated with electrical stimulations to generate or simulate tremor. Orthoses evaluated with test bench experiments show the highest suppression efficacy, the average PSD efficacy of the four evaluations on a test bench being 98.2% (±1.5%). The smallest and most spread efficacies were evaluated with tremor-affected people ranging from 30% (AA) to 98% (PSD). Experiments with healthy subjects showed a range of efficacy from 77% (AA) to 88% (PSD) (Fig. 3).

### Suppression mechanisms and properties

The main components of tremor orthoses are their suppression mechanisms, with the three most common mechanisms currently being electrical motors, pneumatic systems, and viscous/hydraulic configurations. The majority of the suppression orthoses are active with 52% prevalence, of which 73% (38% of all orthoses) rely on electrical motors. The majority (63%) use the electric motor directly (with transmission) to control the human joint. The other motors are used for tendons or alternative mechanisms to transfer the force to the body. Semi-active orthoses account for 29% of the selected papers. Four out of the six semi-active mechanisms are magnetically activated. All magnetically activated semi-active mechanisms are viscous/hydraulic based, with the exception of #10’s electromagnetic friction brake. Passive mechanisms are the least represented suppression type in the literature (19%). No intersection of passive mechanisms was found, since the orthoses use different approaches, like a pneumatic piston-coil (#14) and shear resistance orthosis (#13).Another aspect of the suppression mechanism is its rigidity. Most of the orthoses rely on rigid suppression mechanisms, whereas only five orthoses integrated a non-rigid mechanism (#6, #11, #17, #18, #21).

In comparison to the human muscle, the specific power and energy efficiency of the mechanisms are shown in Table 3. The efficiency of linear motors and pneumatic piston-coil actuators was taken from external literature, not related to the review, since it was not provided in the retrieved publications. The linear motor has the highest specific power with 387.3 W/kg and the highest energy efficiency with 85% [85]. The pneumatic mechanism shows the lowest specific power with 76.3 W/kg and an energy efficiency of 20% [86]. The average specific force of the linear actuators of this review is 228.10 N/kg (±15.40 N/kg), whereas the rotary motor tendon transmission orthosis (#6) was excluded because it uses a transmission system to transfer the motor torque to linear force.

**Table 3:** Specific power, specific force/torque and the efficiency of rotary motors, linear motors and pneumatic piston-coil systems used by the orthoses of this review. Human muscle performance given as reference. Asterisk-tagged values are from external literature, since these data were not provided in the retrieved literature

	Rotary motor with transmission (#1, #4) [38–40, 47–56]	Linear motor (#7) [59]	Pneumatic piston-coil (#8) [60–64]	Human muscle * [83, 84]
Specific power [W/kg]	117.5 (±3.7)	387.30	76.3	50
Specific force/torque	16.7 (±13.8) Nm/kg	212.7 N/kg	243.5 N/kg	20 Nm/kg
Energy efficiency [%]	55.8 (±4.2)	85 * [85]	20 * [86]	35

An active suppression mechanism is characterised by the torque or force. The average torque is 2.3 Nm (±0.73 Nm) excluding one outlier (#4 WOTAS – 8 Nm). The average force of the linear actuators is 15.6 N (±0.6 N), excluding also one outlier (#7 PMLM – 67 N).

Passive and semi-active tremor-suppression orthoses were characterised with the damping coefficient. In damping orthoses, the damping force depends on the velocity and for semi-active mechanisms also on the tuning, the adjustment of the damping coefficient by an additional editable input. One of the two semi-active mechanisms, with given parameters, has a low damping coefficient of 8.9 Ns/m, and can be tuned through adjustment of the magnetic field in the piston-coil for the magnetorheological fluid, to the high damping coefficient of 186.7 Ns/m (#9 MR Damper). The other semi-active mechanism has a larger range from 6.5 Ns/m to 25.0 Ns/m (#12 DVB Orthosis). The passive mechanism #7 PMLM has a constant damping coefficient of 400 Ns/m, whereas #14 Air Dashpot has a damping coefficient of 1800 Ns/m. Two other damping orthoses have a rotary damping coefficient of 2*10^− 3^ Nms/deg. (#13, #16).

### Degrees of freedom

The majority of the orthoses (57%) have one out of four possible DOF of suppression (shoulder excluded), mainly the suppression of the involuntary movement of the EFE (50%) followed by FPS (17%) and WFE (33%). None of the orthoses suppress only WD; however, the orthoses treating only one joint are usually focused on the wrist (47% WFE and WD). The WFE is the most suppressed DOF, whereas the WD is the least supported and also the most locked DOF at the human upper limb. Out of the 13 orthoses providing WFE suppression, 5 also block WD.

### Weight of the orthoses

In general, a higher number of DOF of the device leads to a higher weight of the orthosis. Therefore, the specific weight of the orthoses was investigated. The average specific weight of an orthosis is 418 g/DOF (±270 g/DOF), where the specific weight in the wrist at 231 g/DOF (±45 g/DOF) tends to be less than half of the specific weight in the elbow at 568 g/DOF (±281 g/DOF). The passive orthoses showed on average the lowest specific weight of 248 g/DOF (±35 g/DOF), compared to 415 g/DOF (±242 g/DOF) for active orthoses. The specific weight of the semi-active orthosis considered was by far the highest with 942 g/DOF.

### Informative value of data

**Fig. 4:**
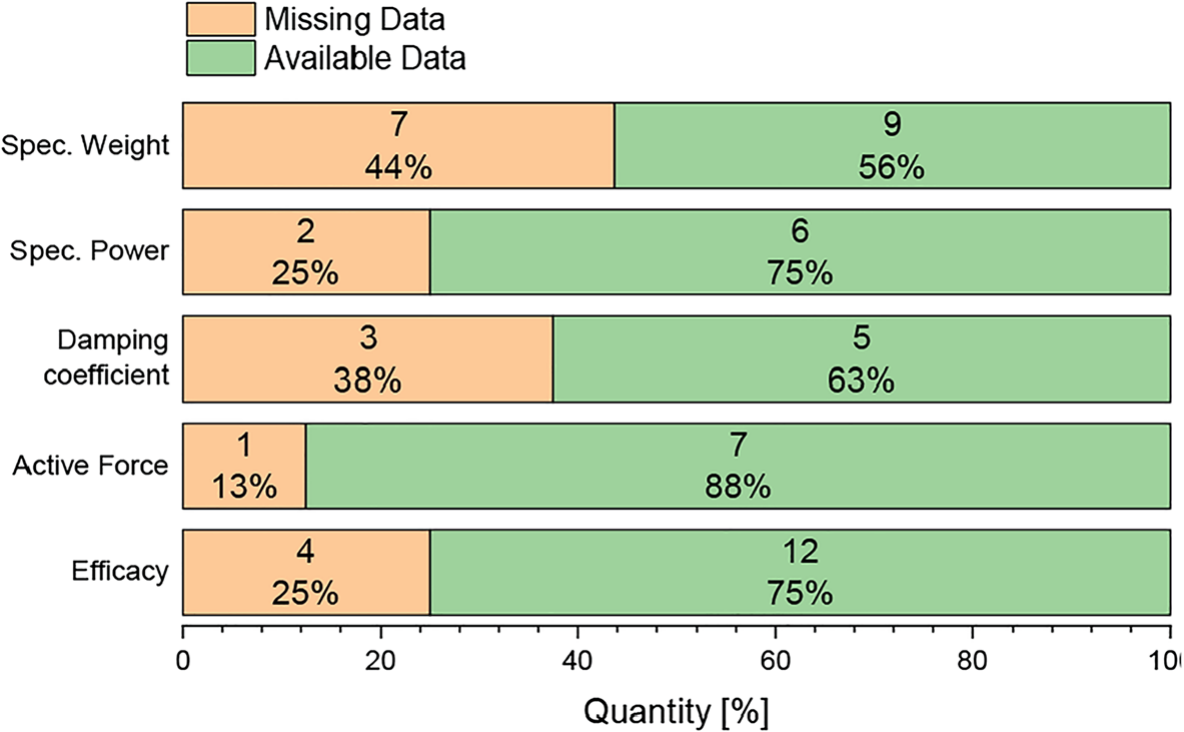
Overview of missing (orange) and available data (green). Shown in percentage and absolute values for specific weight, specific power, damping coefficient, active force, and efficacy

Some of the publications on the compared orthoses did not include all relevant data for this review. Figure 4 shows an overview of missing and available data for the orthoses. The two data sets with the most missing data are the specific weight with 43.8% (7 orthoses) and the damping coefficient with 37.5% (3 orthoses).

## Discussion

### Suppression orthoses efficacy

For this review different efficacy analysis methods were used. The efficacy for most of the considered orthoses was analysed with PSD, whereas the remaining orthoses analysed the beneficial change with the RMS of the tremor amplitude, the average tremor acceleration (AA), or deflection amplitude (AD). Even though different analysis methods may have an impact on the efficacy outcome, it can be assumed that there is a similarity between these values as the analysed variables are co-dependent (distance, acceleration, energy).

In general, the efficacy of suppression is dependent on the mechanical system, but it is also reliant on the sensors, control strategies and human factors. Human factors include the individual adaptation of the subject to the orthoses, the individual characteristics of the disease and/or the individual biomechanical properties, like soft tissue characteristics. The influence of the individual can be seen in Fig. 3. The range of efficacy for tremor subjects is the largest, which can be ascribed to the different interactions of the orthoses with the human. Furthermore, the efficacy varies within one orthosis. One subject out of 10 reached an efficacy of 80% for orthosis #4 WOTAS, whereas the average was 40% (PSD) [52]. Efficacy can also be found to vary within one subject, for instance with different arm postures (orthosis #3) [45]. Furthermore, tremor-affected subjects suffered from different diseases including ET, PD, multiple sclerosis, post-traumatic tremor or mixed tremor. However, most studies focused on ET and PD as the most common movement disorder diseases. In some of the examined literature, subject populations comprised mixed disease diagnoses, whilst other studies considered individuals of only one condition. This disparity led to a large fluctuation in the efficacy of the studied orthoses. The efficacy for studies in tremor subjects ranges from 30% (AA) to 98% (PSD), with an average cross-method efficacy of 63%, which is in the range of medication efficacy (39 – 68% tremor amplitude improvement for ET) [3]. Orthosis #12, DVB Orthosis, reached the highest suppression efficacy (98% PSD). However, the orthosis also suppresses 17% of voluntary movements (magnitude change). Furthermore, tremor subjects usually continue their medication therapy during the studies, which means that the suppression benefit of the orthoses is supplementary to the medication treatment. Neither pharmaceutical long-term studies nor orthotic long-term studies were sufficiently performed for comparison. However, tremor intensity increases over the progress of the disease, whereas the performed studies indicate that the efficacy of medication decreases over time. We assume that the efficacy performance of orthoses increases with more severe tremor, because most tremor suppression mechanisms reduce tremor to a certain baseline and are thereby independent from the severity of the tremor. It is unclear how tremor reacts to long-term mechanical suppression. Increase of tremor by muscle training due to resistance force by the orthosis, as well as decrease of tremor by the mechanical interruption of the sensory feedback of the nervous system, are possible effects. Furthermore, unknown side-effects and phenomena could appear and/or intensify, such as the Distal to Proximal Tremor Shift.

For the experiments with healthy subjects, tremorous movements were stimulated by either electricity or the subject imitating the movement. The properties of the soft tissues were included in these experiments, but it is unknown how comparable tremor simulation and stimulation are to condition-induced tremor. Furthermore, the retrieved studies used a small number of subjects, most often only one subject; as a result, the experiments are more a proof of concept than representative of a complete quantitative study on efficacy. Future evaluations need to aim for clinical trials with a statistically reliable number of subjects, while at the same time a placebo group should be included. The development of placebo-controlled trials is necessary because the mind-set might have a positive impact on the human control system, which reinforces the human motor control and reduces tremor. Placebo-controlled trials are a challenge, since a placebo orthosis itself will always have an impact on the wearer, as even just the weight of the placebo orthosis has a tremor suppression effect. Due to technical limitations, an orthosis will always have some resistance force and interaction with the wearer, especially in passive but also in semi-active and even active systems. A suitable approach for a placebo-controlled trial needs to be developed to account for the placebo effect.

Applying an external force to the human musculoskeletal system introduces further difficulties, as the involuntary muscle activity applies the force directly onto the skeletal system and therefore requires the orthosis to suppress this movement through the soft tissues (skin, fat, muscles, etc.). The stiffness between an orthosis and the human body is a key factor for tremor suppression as joint movement areas, surface tendons and surface nerves or vessels need to be avoided to prevent harm when applying force to the human system. Furthermore, the stiffness of the tissues increases with the applied pressure [32, 55]. Besides the flux of force, soft tissues also influence sensory information, which are needed among other for a closed feedback loop. In order to optimise the system, soft tissues can be modelled with viscoelastic or elastic properties [87]. The viscoelastic soft tissue model acts as a parallel non-linear spring-damper-system in the flux of force (Voight element) [88].

Although most test bench experiments used recorded tremor data, they use a simplified and idealised system, not considering the potential influence of soft tissues or human factors. These simplifications, as expected, lead to the highest efficacies, up to 99.8% (PSD) with a small variation. The distribution of the efficacies shows the impact of soft tissues and the human factors, including the special biomechanics of the diseases. The biggest challenge for the design of an effective orthosis is the human-machine interface. In this field improvements are necessary, even though there is a mechanical limit due to soft tissues.

In order to put the orthosis efficacy in relation, the efficacy of other alternative and supplementary treatments is of interest. The management of tremor through functional electrical stimulation by Rocon’s group reduced the tremor power by 52% (PSD), which was their second approach after the orthosis WOTAS (#4) [25]. However, rapid fatigue of the stimulated muscle, discomfort due to strong stimulation, and interference with voluntary movements are drawbacks affecting the wearers’ acceptance and the long-term effectiveness of functional electrical stimulation. The third approach of Rocon was sensory electrical stimulation, muscle stimulation below the functional activation threshold, which reached 52% reduction of tremor power on average (with individual parameter optimisation), which is in both cases an improvement compared to the 40% WOTAS (#4) efficacy. The variability in suppression in sensory electrical stimulation was high, with one third of the patients not responding to the therapy. Furthermore, over long-term use the efficacy may be impaired by neural adaptation [89]. Dideriksen et al. showed that the state-of-the-art in suppression of tremor using electrical stimulation does not transcend 67% on average [89], whereas tremor suppression orthoses have the potential to achieve higher suppression magnitudes as the semi-active DVB Orthosis (#12) did, with 98%.

### Suppression mechanisms and properties

Active mechanisms are theoretically more advantageous than passive mechanisms because they do not generate resistance force for voluntary movements of the user. In reality, correct recognition of a user’s voluntary and involuntary movements by the control system presents a great challenge. The majority of the suppression orthoses rely on electric motors which need a transmission system in order to actuate the limb in the correct manner. These transmission systems are associated with additional weight and installation space, which results in a bulkier mechanism. Additionally, a high specific power and energy efficiency are beneficial for the orthosis’ performance and weight. The rotary motor with transmission exceeds the human muscle regarding the specific power and efficiency. Even though the requirements of the actuation system vary from those of the human muscle, the human muscle can serve as a point of orientation. Orthoses with a linear actuator need to be evaluated individually because the attachment of the orthosis to the human limb affects the resulting torque in the limb joint.

Semi-active mechanisms can minimise the resistance for voluntary movements and maximise it for involuntary movements. Most of the semi-active mechanisms use a magnetic field and magnetorheological fluid to tune the suppression system. Like the linear actuators, a quantitative comparison of the linear damper is difficult because the orthoses are attached differently to the limb. Their rotary damping coefficient is unknown and may have different proportions to that of the linear damping coefficient. The passive suppression mechanisms are not tuneable and are therefore lightweight. With a constant damping coefficient there is a compromise between suppressing voluntary movements in some parts and allowing involuntary movements in others. However, the advantage is the simplicity and lightweight design of such a system. Despite this, only a few papers about passive orthoses have been published, probably because of the disadvantages of such simple mechanisms.

The soft tissue pressure threshold and pressure discomfort threshold differ for each individual subject, through both inter-subject and intra-subject variation. In general, areas of the human body show a lower or higher pressure pain and discomfort threshold at different locations [90]. It is therefore important where and how an orthosis is attached to the human body. Skin properties may also vary within a day and with age. Furthermore, mechanical properties of the skin like stiffness, friction and thickness change with its hydration and climate conditions [91, 92]. A potential source of harm for the skeletal system is a misalignment with rigid actuators and structures, for which the inter-subject anatomical variation would consequently require an orthosis, which can adapt to a user’s anthropometry. A further safety hazard is the misalignment of the orthoses hinge and the human joint, where typically a slight human joint axis displacement is observed during rotation, such as the wrist [93].

Soft mechanisms show a high potential for wearable robotic devices, as they are less bulky and especially visually more appealing. Current users of conventional rigid robotic orthoses claim that these are too bulky, which can lead to negative effects in activities of daily living or in the worst case to social exclusion [52]. Out of the 21 retrieved orthoses, only 5 are soft orthoses without rigid structures or a suppression mechanism. Among the 16 prototypes, three orthoses propose a soft system. Veale et al. propose to direct research towards compliant robotic orthoses [94].

### Suppression orthoses efficacy

The efficacy of the suppression orthosis indicates how well the orthosis counteracts tremor. The performance of the prototypes was evaluated either through test bench experiments, on tremor-affected patients or on healthy subjects, the latter of whom were treated with electrical stimulations to generate or simulate tremor. Orthoses evaluated with test bench experiments show the highest suppression efficacy, the average PSD efficacy of the four evaluations on a test bench being 98.2% (±1.5%). The smallest and most spread efficacies were evaluated with tremor-affected people ranging from 30% (AA) to 98% (PSD). Experiments with healthy subjects showed a range of efficacy from 77% (AA) to 88% (PSD) (Fig. 3).

### Suppression mechanisms and properties

The main components of tremor orthoses are their suppression mechanisms, with the three most common mechanisms currently being electrical motors, pneumatic systems, and viscous/hydraulic configurations. The majority of the suppression orthoses are active with 52% prevalence, of which 73% (38% of all orthoses) rely on electrical motors. The majority (63%) use the electric motor directly (with transmission) to control the human joint. The other motors are used for tendons or alternative mechanisms to transfer the force to the body. Semi-active orthoses account for 29% of the selected papers. Four out of the six semi-active mechanisms are magnetically activated. All magnetically activated semi-active mechanisms are viscous/hydraulic based, with the exception of #10’s electromagnetic friction brake. Passive mechanisms are the least represented suppression type in the literature (19%). No intersection of passive mechanisms was found, since the orthoses use different approaches, like a pneumatic piston-coil (#14) and shear resistance orthosis (#13).

Another aspect of the suppression mechanism is its rigidity. Most of the orthoses rely on rigid suppression mechanisms, whereas only five orthoses integrated a non-rigid mechanism (#6, #11, #17, #18, #21).

In comparison to the human muscle, the specific power and energy efficiency of the mechanisms are shown in Table 3. The efficiency of linear motors and pneumatic piston-coil actuators was taken from external literature, not related to the review, since it was not provided in the retrieved publications. The linear motor has the highest specific power with 387.3 W/kg and the highest energy efficiency with 85% [85]. The pneumatic mechanism shows the lowest specific power with 76.3 W/kg and an energy efficiency of 20% [86]. The average specific force of the linear actuators of this review is 228.10 N/kg (±15.40 N/kg), whereas the rotary motor tendon transmission orthosis (#6) was excluded because it uses a transmission system to transfer the motor torque to linear force.

An active suppression mechanism is characterised by the torque or force. The average torque is 2.3 Nm (±0.73 Nm) excluding one outlier (#4 WOTAS – 8 Nm). The average force of the linear actuators is 15.6 N (±0.6 N), excluding also one outlier (#7 PMLM – 67 N).

Passive and semi-active tremor-suppression orthoses were characterised with the damping coefficient. In damping orthoses, the damping force depends on the velocity and for semi-active mechanisms also on the tuning, the adjustment of the damping coefficient by an additional editable input. One of the two semi-active mechanisms, with given parameters, has a low damping coefficient of 8.9 Ns/m, and can be tuned through adjustment of the magnetic field in the piston-coil for the magnetorheological fluid, to the high damping coefficient of 186.7 Ns/m (#9 MR Damper). The other semi-active mechanism has a larger range from 6.5 Ns/m to 25.0 Ns/m (#12 DVB Orthosis). The passive mechanism #7 PMLM has a constant damping coefficient of 400 Ns/m, whereas #14 Air Dashpot has a damping coefficient of 1800 Ns/m. Two other damping orthoses have a rotary damping coefficient of 2*10^− 3^ Nms/deg. (#13, #16).

### Degrees of freedom

For the ergonomics of an orthosis, the DOF are relevant. A locked DOF restricts the natural workspace of the user. Such restrictions lead to discomfort and limited acceptance by the orthosis user. The review of the current literature shows that many orthoses lock the wrist deviation, a movement which can be compensated by the user’s shoulder movement, but this can lead to an overload and/or false posture of the shoulder in everyday usage [46]. Most orthoses concentrate on suppressing tremor in one DOF, like the elbow or wrist (flexion and extension), whereas only one group investigated the biomechanical kinematics of tremor (#4 WOTAS) [31]. However, for postural tremor of ET patients, the WFE and FPS are the kinematics with the highest measured tremorous impact in the upper limb [30]. Many orthoses considered the WFE but only seven orthoses suppress involuntary movements of the FPS, a reason being the mechanical complexity of FPS intervention [32]. Regardless, the exact biomechanical processes and origins of tremor are still unclear and there is a need for further investigations to achieve an optimal suppression [95]. In general, it can be assumed that WFE and FPS suppression are sufficient for an adequate physical intervention in tremor because those are the most affected DOF in PD, ET and cerebellar tremor [96].

### Weight of the orthoses

The weight of a wearable orthosis is also an essential factor for its ergonomics. Additional weight at the arm can lead to muscle fatigue and discomfort and is one of the most important design factors [97]. The average weight of an orthosis which suppresses involuntary movements in the elbow is 580 g, corresponding to a 19% increase in the weight of an average user’s arm (average forearm weight of 2.3% and arm weight of 5% from average body weight of 62 kg [98, 99]). Comparatively, an orthosis attached to the forearm and/or hands may average 231 g for a 16% increase in the weight of the forearm. In both calculations, the weight of the energy source and control unit were not considered as these additional components will further increase the total weight of the orthoses. To keep the total weight at the arm, these components could be attached decentralized, e.g. like a backpack, instead of being integrated into the orthoses. Furthermore, the results of the specific weight show a high standard deviation because it is influenced by many factors, like the actuation system, cuff system, and lightweight design and development status, respectively. Too heavy orthoses can be an exclusion criterion for the wearer’s everyday usage. Users of the WOTAS (#4) claimed that the orthosis is too bulky and leads to muscle fatigue [52]. This led to a cessation of the research on this wearable orthosis as it was not an acceptable solution for the patients [100]. Instead, the group investigated taking a different approach (functional electrical stimulation). This shows the importance of the weight and the need for improvement.

### Number of publications over time

With one exception, all publications retrieved in this review were published in the last two decades, with the majority having been written in the last 8 years. Robotic technology in general is progressing, leading to an increasing number of sophisticated wearable and portable devices. Robotic technology is a growing field of research especially for rehabilitation and biomedical engineering. Three types of suppression strategies were observed; active, semi-active and passive, with active suppression orthoses found to be the most prevalent. Because of the inherent differences between the categories, it is difficult to perform a cross-comparison of the orthoses. An analysis and comparison of special mechanical or ergonomic characteristics can only occur qualitatively as, due to the low sample size and the nominal scale of the data, quantitative analysis is restricted.

### Future research and development

Most of the prototypes did not reach the market due to the low wearability, leading to a lack of acceptance by the user. Here, wearability is the comfort and ergonomics in contrast to the performance. The high weight and the rigid structures lead to low ergonomics and comfort. Especially elder patients are more sensitive to weight due to sarcopenia, the degenerative loss of skeletal muscle mass associated with aging. Furthermore, older people often have a negative attitude towards technology, especially towards gerontechnology [101]; therefore a high wearability and an unobtrusive design are required improvements.

In order to develop appropriate tremor-suppression orthoses, the biomechanics of the tremorous movement need to be characterized, as proposed by Charles et al. [95]. Further investigations for the human-machine interface are needed, to improve the connection of a wearable device to the human body with an ideal force transmission. For this, an improvement in the understanding of tremorous movements and influencing factors of soft tissues are crucial.. Future research probably needs to focus on soft suppression mechanisms with improved efficacy for tremor suppression to attain higher rates of patient acceptance. To this end, a future orthosis needs to be less obtrusive and more visually appealing, as well as to incorporate more biomimetic design features, inspired by nature’s functions and mechanisms. Such suppression orthoses could rely on proposed mechanisms like hydraulically amplified soft actuators, Peano-HASEL [102], dielectric elastomers, low melting point alloys, shape memory alloys or other mechanisms reviewed by Chen and Pei [103]. This higher patient acceptance combined with improved efficacy could be achieved by the improved wearability along with such soft mechanisms and the new possibilities of an unobtrusive design. A new orthosis with a soft suppression system and improved suppression efficacy needs to have enough variability to accommodate all patients and different tremor types. Such an orthosis could be used in future to investigate the effect of such tremor-suppression orthosis on the subject and its involuntary movement in short- and long-term use, like the Distal to Proximal Tremor Shift phenomenon.

## Conclusion

This paper reviews the state-of-the-art and persisting trends of tremor-suppression wearable orthoses within the literature search. Twenty-one orthoses were identified through a literature search, with distinct suppression types (passive, semi-active, and active), and further classified by their status of development (concepts and prototypes). Their different mechanical and ergonomic properties were mapped and compared, including DOF, weight, suppression properties, and efficacy. This review will help researchers to gain a deeper understanding of the state-of-the-art and weaknesses of the identified suppression orthoses.

This review shows that the majority of the tremor-suppression orthoses are bulky and have rigid structures, adding weight of about 20% to the arm, not including the energy source and control unit. The efficacy of involuntary movement attenuation with tremorous patients was found to be 63% in the cross-method average, which shows a better average performance than the most common treatment - medication (23 to 68%) - and was in most cases supplementary to the benefit of the medication. The manipulation of movement by electrical stimulation achieves a comparable average suppression efficacy of 67%. Invasive methods (DBS) achieve 90% reduction and the emerging and less invasive high intensity focused ultrasound 55% tremor suppression efficacy. The orthotic approach has the advantage that it is a non-invasive method as opposed to DBS and high intensity focused ultrasound, and can also be used as a supplement to invasive methods.

This performance of future orthoses will be further improved with an optimised human-machine interface, a better understanding of the tremorous movement pattern and improved suppression mechanism. Many orthoses are unwieldy since they are constraining the natural workspace of the wearer. Furthermore, visually unappealing orthoses are often rejected by patients as they may be considered to lead to social exclusion.

The main challenge of the design of tremor-cancelling orthoses is the combination of a high tremor suppression efficacy mechanism with good ergonomics, leading to high acceptance especially by older people. More precisely, the challenge to design an optimal tremor-cancelling orthosis is the development of a soft, non-cumbersome, compact, powerful, lightweight, and direct-driven suppression system with an appropriate design to increase the wearers’ acceptance. All these characteristics are obviously very challenging and have not been achieved by existing systems, which clearly indicates the necessity for the development of a novel suppression orthosis with minimal burden for the patient and improved suppression efficacy. In this review, some concepts using lightweight structures and smart textiles, such as the unobtrusive appearance and soft mechanism of the Piezoelectric Fibre Glove (#17), demonstrate a promising route to address these shortcomings. To reach that design goal, further research and development is required in this field.

## Data Availability

All data generated or analysed during this study are included in this published article.

## Abbreviations

AA: Average tremor acceleration amplitude [m/s^2^; deg./s^2^]
AD: Average tremor deflection amplitude [deg/s^2^]
DBS: Deep brain stimulation
DOF: Degree of freedom
EFE: Elbow flexion/extension
ET: Essential tremor
FPS: Forearm pronation/supination
PD: Parkinson’s disease
PSD: Power spectral density of tremor [rad^2^/s^3^/Hz]
RMS: Root mean square of tremor acceleration amplitude [m/s^2^]
WD: Wrist radial/ulnar deviation
WFE: Wrist flexion/extension

## References

[1] Deuschl G, Bain P, Brin M (1998). Consensus statement of the Movement Disorder Society on Tremor. Mov Disord.

[2] Bötzel K, Tronnier V, Gasser T (2014). The differential diagnosis and treatment of tremor. Dtsch Arztebl Int.

[3] Elble R, Deuschl G (2011). Milestones in tremor research. Mov Disord.

[4] Ellrichmann G (2007). Vorkommen und Wertigkeit von Oberfrequenzen in der 24-Stunden-Elektromyographie und Accelerometrie. Doctoral dissertation.

[5] Raethjen J, Lindemann M, Schmajohann H, Wenzelburger R, Pfister G, Deuschl G (2000). Multiple oscillators are causing parkinsonian and essential tremor. Mov Disord.

[6] Louis ED, Ferreira JJ (2010). How common is the most common adult movement disorder? Update on the worldwide prevalence of essential tremor. Mov Disord.

[7] de Rijk MC, Breteler MM, Graveland GA, Ott A, Grobbee DE, van der Meché FG (1995). Prevalence of Parkinson’s disease in the elderly: the Rotterdam study. Neurology.

[8] Lopez-de-Ipiña K, Bergareche A, de la Riva P, Faundez-Zanuy M, Calvo PM, Roure J (2014). Automatic non-linear analysis of non-invasive writing signals, applied to essential tremor. J Appl Log.

[9] Eurostat (2018). Population structure and ageing - Eurostat.

[10] Grimaldi G, Manto M (2010). “Old” and emerging therapies of human tremor. Clin Med Insights Ther.

[11] Miller KM, Okun MS, Fernandez HF, Jacobson CE, Rodriguez RL, Bowers D (2007). Depression symptoms in movement disorders: comparing Parkinson’s disease, dystonia, and essential tremor. Mov Disord.

[12] Jankovic J (2008). Parkinson’s disease: clinical features and diagnosis. J Neurol Neurosurg Psychiatry.

[13] Cohen O, Pullman S, Jurewicz E, Watner D, Louis ED (2003). Rest tremor in patients with essential tremor: prevalence, clinical correlates, and electrophysiologic characteristics. Arch Neurol.

[14] Gerlach M, Reichmann H, Riederer P, Dietmaier O, Götz W, Laux G (2007). Die Parkinson-Krankheit.

[15] Rana AQ, Chou KL (2015). Essential tremor in clinical practice.

[16] Ruonala V, Meigal A, Rissanen SM, Airaksinen O, Kankaanpää M, Karjalainen PA (2014). EMG signal morphology and kinematic parameters in essential tremor and Parkinson’s disease patients. J Electromyogr Kinesiol.

[17] O’Connor RJ, Kini MU (2011). Non-pharmacological and non-surgical interventions for tremor: a systematic review. Park Relat Disord.

[18] Diaz NL, Louis ED (2010). Survey of medication usage patterns among essential tremor patients: movement disorder specialists vs. general neurologists. Park Relat Disord.

[19] Hariz MI, Rehncrona S, Quinn NP, Speelman JD, Wensing C (2008). Multicenter study on deep brain stimulation in Parkinson’s disease: an independent assessment of reported adverse events at 4 years. Mov Disord.

[20] Koller WC (1986). Pharmacologic treatment of parkinsonian tremor. Arch Neurol.

[21] Koller WC, Lyons KE, Wilkinson SB, Troster AI, Pahwa R (2001). Long-term safety and efficacy of unilateral deep brain stimulation of the thalamus in essential tremor. Mov Disord.

[22] Sasso E, Perucca E, Fava R, Calzetti S (1990). Primidone in the long-term treatment of essential tremor: a prospective study with computerized quantitative analysis. Clin Neuropharmacol.

[23] Chang JW, Park CK, Lipsman N, Schwartz ML, Ghanouni P, Henderson JM (2018). A prospective trial of magnetic resonance–guided focused ultrasound thalamotomy for essential tremor: results at the 2-year follow-up. Ann Neurol.

[24] Elias WJ, Lipsman N, Ondo WG, Ghanouni P, Kim YG, Lee W (2016). A randomized trial of focused ultrasound thalamotomy for essential tremor. N Engl J Med.

[25] Gallego JÁ, Rocon E, Belda-Lois JM, Pons JL (2013). A neuroprosthesis for tremor management through the control of muscle co-contraction. J Neuroeng Rehabil.

[26] Keus SHJ, Munneke M, Nijkrake MJ, Kwakkel G, Bloem BR (2009). Physical therapy in Parkinson’s disease: evolution and future challenges. Mov Disord.

[27] Pons JL (2008). Wearable robots : biomechatronic exoskeletons.

[28] Manto M, Rocon E, Pons J, Belda JM, Camut S (2007). Evaluation of a wearable orthosis and an associated algorithm for tremor suppression. Physiol Meas.

[29] Hellwig B, Mund P, Schelter B, Guschlbauer B, Timmer J, Lücking CH (2009). A longitudinal study of tremor frequencies in Parkinson’s disease and essential tremor. Clin Neurophysiol.

[30] Geiger DW (2014). Characterization of postural tremor in essential tremor using a seven-degree-of-freedom model.

[31] Belda-Lois JM, Rocon E, Sanchez-Lacuesta JJ, Ruiz AF, Pons JL (2005). Estimation of biomechanical characteristics of tremorous movements based on gyroscopes. Assist Technol-from Virtuality Real.

[32] Rocon E, Pons JL (2011). Exoskeletons in Rehabilitation Robotics. Springer Tracts in Advanced Robotics.

[33] Schiele A, van der Helm FCT (2006). Kinematic design to improve ergonomics in human machine interaction. IEEE Trans Neural Syst Rehabil Eng.

[34] Lakie M, Vernooij CA, Osborne TM, Reynolds RF (2012). The resonant component of human physiological hand tremor is altered by slow voluntary movements. J Physiol.

[35] Lakie M, Vernooij CA, Osler CJ, Stevenson AT, Scott JPR, Reynolds RF (2015). Increased gravitational force reveals the mechanical, resonant nature of physiological tremor. J Physiol.

[36] Biddiss E, Beaton D, Chau T (2007). Consumer design priorities for upper limb prosthetics. Disabil Rehabil Assist Technol.

[37] Perry JC, Rosen J, Burns S (2007). Upper-limb powered exoskeleton design. IEEE/ASME Trans Mechatron.

[38] Herrnstadt G, Menon C (2017). Elbow orthosis for tremor suppression – a torque based input case. Bioinformatics and biomedical engineering IWBBIO 2017 lecture notes in computer science.

[39] Herrnstadt G, Menon C (2016). Voluntary-driven elbow orthosis with speed-controlled tremor suppression. Front Bioeng Biotechnol.

[40] Herrnstadt G, Menon C (2016). Admittance-based voluntary-driven motion with speed-controlled tremor rejection. IEEE/ASME Trans Mechatron.

[41] Seki M, Matsumoto Y, Ando T, Kobayashi Y, Iijima H, Nagaoka M (2011). The weight load inconsistency effect on voluntary movement recognition of essential tremor patient. 2011 IEEE international conference on Robotics and biomimetics, ROBIO 2011.

[42] Ando T, Watanabe M, Nishimoto K, Matsumoto Y, Seki M, Fujie MG (2012). Myoelectric-controlled exoskeletal elbow robot to suppress essential tremor: extraction of elbow flexion movement using STFTs and TDNN. J Robot Mechatron.

[43] Ando T, Watanabe M, Fujie MG (2009). Extraction of voluntary movement for an EMG controlled exoskeltal robot of tremor patients. 2009 4th international IEEE/EMBS conference on neural engineering, NER ‘09.

[44] Seki M, Matsumoto Y, Ando T, Kobayashi Y, Fujie MG, Iijima H (2011). Development of robotic upper limb orthosis with tremor suppressiblity and elbow joint movability. Conference Proceedings - IEEE International Conference on Systems, Man and Cybernetics.

[45] Matsumoto Y, Seki M, Ando T, Kobayashi Y, Nakashima Y, Iijima H (2013). Development of an exoskeleton to support eating movements in patients with essential tremor. J Robot Mechatron.

[46] Matsumoto Y, Amemiya M, Kaneishi D, Nakashima Y, Seki M, Ando T (2014). Development of an elbow-forearm interlock joint mechanism toward an exoskeleton for patients with essential tremor. IEEE International Conference on Intelligent Robots and Systems. Institute of Electrical and Electronics Engineers Inc.

[47] Rocon E, Pons JL (2011). Upper limb exoskeleton for tremor suppression: validation. Vol. 69, Springer Tracts in Advanced Robotics.

[48] Rocon E, Pons JL (2011). Upper limb exoskeleton for tremor suppression: cognitive HR interaction. Springer tracts in advanced Robotics.

[49] Belda-Lois J, Martinez-Reyero A, Castillo A, Rocon E, Pons J, Loureiro R (2007). Controllable mechanical tremor reduction. Assessment of two orthoses. Technol Disabil.

[50] Rocon E, Ruiz AF, Brunetti F, Pons JL, Belda-Lois JM, Sánchez-Lacuesta JJ (2006). On the use of an active wearable exoskeleton for tremor suppression via biomechanical loading. Proceedings - IEEE International Conference on Robotics and Automation.

[51] Rocon E, Ruiz AF, Pons JL, Belda-Lois JM, Sánchez-Lacuesta JJ (2005). Rehabilitation robotics: a wearable exo-skeleton for tremor assessment and suppression. Proceedings - IEEE International Conference on Robotics and Automation.

[52] Rocon E, Gallego JA, Belda-Lois JM, Pons JL, Sanfeliu A, Ferre M, Armada MA (2014). Assistive robotics as alternative treatment for tremor. Vol. 252, Advances in Intelligent Systems and Computing.

[53] Rocon E, Belda-Lois J, Sanchez-Lacuesta J, Pons J (2004). Pathological tremor management: modelling, compensatory technology and evaluation. Technol Disabil.

[54] Rocon E, Belda-Lois JM, Ruiz AF, Manto M, Moreno JC, Pons JL (2007). Design and validation of a rehabilitation robotic exoskeleton for tremor assessment and suppression. IEEE Trans Neural Syst Rehabil Eng.

[55] Rocon E, Pons JL (2011). Upper limb exoskeleton for tremor suppression: Physical HR interaction. Vol. 69, Springer Tracts in Advanced Robotics.

[56] Rocon E, Manto M, Pons J, Camut S, Belda JM (2007). Mechanical suppression of essential tremor. Cerebellum.

[57] Huen D, Liu J, Lo B (2016). An integrated wearable robot for tremor suppression with context aware sensing. 2016 IEEE 13th International Conference on Wearable and Implantable Body Sensor Networks (BSN).

[58] Zhou Y, Naish MD, Jenkins ME, Trejos AL (2017). Design and validation of a novel mechatronic transmission system for a wearable tremor suppression device. Rob Auton Syst.

[59] Zamanian AH, Richer E (2017). Adaptive disturbance rejection controller for pathological tremor suppression with permanent magnet linear motor. ASME 2017 dynamic systems and control conference. Tysons.

[60] Taheri B, Case D, Richer E (2014). Robust controller for tremor suppression at musculoskeletal level in human wrist. IEEE Trans Neural Syst Rehabil Eng.

[61] Taheri B, Case D, Richer E (2011). Active tremor estimation and suppression in human elbow joint. ASME 2011 dynamic systems and control conference and Bath/ASME symposium on fluid power and motion control.

[62] Taheri B, Case D, Richer E (2015). Adaptive suppression of severe pathological tremor by torque estimation method. IEEE/ASME Trans Mechatron.

[63] Taheri B, Case D, Richer E (2013). Theoretical development and experimental validation of an adaptive controller for tremor suppression at musculoskeletal level. ASME 2013 Dynamic Systems and Control Conference.

[64] Taheri B (2013). Real-time pathological tremor identification and suppression in human arm via active orthotic devices.

[65] Case D, Taheri B, Richer E (2013). Multiphysics modeling of magnetorheological dampers. Int J Multiphys.

[66] Case D, Taheri B, Richer E (2013). Design and characterization of a small-scale magnetorheological damper for tremor suppression. IEEE/ASME Trans Mechatron.

[67] Case D, Taheri B, Richer E (2015). Active control of MR wearable robotic orthosis for pathological tremor suppression. ASME 2015 dynamic systems and control conference.

[68] Case D, Taheri B, Richer E (2015). A lumped-parameter model for adaptive dynamic MR damper control. IEEE/ASME Trans Mechatron.

[69] Case D, Taheri B, Richer E (2014). Dynamical modeling and experimental study of a small-scale magnetorheological damper. IEEE/ASME Trans Mechatron.

[70] Case D, Taheri B, Richer E (2011). Dynamic magnetorheological damper for orthotic tremor suppression. HUIC Mathematics & Engineering.

[71] Herrnstadt G, Menon C (2013). On-off tremor suppression orthosis with electromagnetic brake. Int J Mech Eng Mechatron.

[72] Kalaiarasi A, Kumar LA (2018). Sensor based portable tremor suppression device for stroke patients. Acupunct Electrother Res.

[73] Loureiro RCV, Belda-Lois JM, Lima ER, Pons JL, Sanchez-Lacuesta JJ, Harwin WS (2005). Upper limb tremor suppression in ADL via an orthosis incorporating a controllable double viscous beam actuator. Proceedings of the 2005 IEEE 9th international conference on Rehabilitation Robotics.

[74] Kotovsky J, Rosen MJ (1998). A wearable tremor-suppression orthosis. J Appl Phys.

[75] Takanokura M, Sugahara R, Miyake N, Ishiguro K, Muto T, Sakamoto K (2011). Upper-limb orthoses implemented with air dashpots for suppression of pathological tremor in daily activites. ISB conference July 2011. Brussel.

[76] Katz R, Buki E, Zacksenhouse M (2017). Attenuating tremor using passive devices. Vol. 242, Studies in health technology and informatics.

[77] Swallow LM, Luo JK, Siores E, Patel I, Dodds D (2008). A piezoelectric fibre composite based energy harvesting device for potential wearable applications. Smart Mater Struct.

[78] Swallow L, Siores E (2009). Tremor suppression ssing smart textile fibre systems. J Fiber Bioeng Informatics.

[79] Kelley CR, Kauffman JL, Bar-Cohen Y (2017). Exploring dielectric elastomers as actuators for hand tremor suppression.

[80] Chuanasa J, Songschon S (2011). Anti-shaker simulation for arm tremor. Circuits, Syst Simul.

[81] Li JQ, Zang XZ, Zhao J (2010). Tremor suppression method via magnetorheological damper and fuzzy neural network control. J Donghua Univ.

[82] Shamroukh M, Kalimullah IQ, Chacko A, Barlingay SS, Kalaichelvi V, Chattopadhyay AB (2017). Evaluation of control strategies in semi-active orthosis for suppression of upper limb pathological tremors. 2017 International Conference on Innovations in Electrical, Electronics, Instrumentation and Media Technology (ICEEIMT).

[83] Hollerbach JM, Hunter I, Ballantyne J (1991). A comparative analysis of actuator technologies for robotics. Vol. 2, The Robotics Review.

[84] Hunter IW, Lafontaine S (1992). A comparison of muscle with artificial actuators. Technical Digest IEEE Solid-State Sensor and Actuator Workshop.

[85] Stock A (2010). Comparing performance and efficiency of linear motors, ball screw, and rack-and-pinion drives.

[86] Yusop MYM (2006). Energy saving for pneumatic actuation using dynamic model prediction.

[87] Maurel W (1999). 3D modeling of the human upper limb including the biomechanics of joints, muscles and soft tissues. Biomechanics.

[88] Fung Y-C (1993). Bioviscoelastic solids. Biomechanics.

[89] Dideriksen JL, Laine CM, Dosen S, Muceli S, Rocon E, Pons JL (2017). Electrical stimulation of afferent pathways for the suppression of pathological tremor. Front Neurosci.

[90] Byström S, Hall C, Welander T, Kilbom Å (1995). Clinical disorders and pressure-pain threshold of the forearm and hand among automobile assembly line workers. J Hand Surg Am.

[91] Hendriks CP, Franklin SE (2010). Influence of surface roughness, material and climate conditions on the friction of human skin. Tribol Lett.

[92] Dąbrowska AK, Adlhart C, Spano F, Rotaru G-M, Derler S, Zhai L (2016). In vivo confirmation of hydration-induced changes in human-skin thickness, roughness and interaction with the environment. Biointerphases.

[93] Neu CP, Crisco JJ, Wolfe SW (2001). In vivo kinematic behavior of the radio-capitate joint during wrist flexion-extension and radio-ulnar deviation. J Biomech.

[94] Veale AJ, Xie SQ (2016). Towards compliant and wearable robotic orthoses: a review of current and emerging actuator technologies. Med Eng Phys.

[95] Charles SK, Geiger DW, Davidson AD, Pigg AC, Curtis CP, Allen BC (2017). Toward quantitative characterization of essential tremor for future tremor suppression. IEEE Int Conf Rehabil Robot.

[96] Elble RJ, Koller WC (1990). Tremor (Johns Hopkins series in contemporary medicine and public health).

[97] Hofmann UAT, Butzer T, Lambercy O, Gassert R (2018). Design and evaluation of a bowden-cable-based remote actuation system for wearable robotics. IEEE Robot Autom Lett.

[98] Clauser CE, McConville JT, Young JW (1969). Weight, volume, and center of mass of segments of the human body. National Technical Information Service.

[99] Walpole SC, Prieto-Merino D, Edwards P, Cleland J, Stevens G, Roberts I (2012). The weight of nations: an estimation of adult human biomass. BMC Public Health.

[100] Rocon E, Gallego JÁ, Belda-Lois JM, Benito-León J, Luis Pons J (2012). Biomechanical loading as an alternative treatment for tremor: a review of two approaches. Tremor Other Hyperkinet Mov.

[101] Yusif S, Soar J, Hafeez-Baig A (2016). Older people, assistive technologies, and the barriers to adoption: a systematic review. Int J Med Inform.

[102] Kellaris N, Gopaluni Venkata V, Smith GM, Mitchell SK, Keplinger C (2018). Peano-HASEL actuators: muscle-mimetic, electrohydraulic transducers that linearly contract on activation. Sci Robot.

[103] Chen D, Pei Q (2017). Electronic muscles and skins: a review of soft sensors and actuators. Chem Rev.

